# Kunxian Capsule for Rheumatoid Arthritis: Inhibition of Inflammatory Network and Reducing Adverse Reactions Through Drug Matching

**DOI:** 10.3389/fphar.2020.00485

**Published:** 2020-04-17

**Authors:** Yujun Tang, Yi Zhang, Lin Li, Zhijun Xie, Chengping Wen, Lin Huang

**Affiliations:** College of Basic Medical Science, Zhejiang Chinese Medical University, Hangzhou, China

**Keywords:** Kunxian Capsule, rheumatoid arthritis, pharmacological mechanism, side effects alleviation, drug matching, bioinformatics

## Abstract

*Tripterygium wilfordii Hook.f* and *Tripterygium hypoglaucum (H.Lév.) Hutch* is effective herbs to prevent aggravation of Rheumatoid arthritis (RA). However, both of them show severe side effects in the reproductive system and other systems. Kunxian Capsule (KX), a Traditional Chinese Medicine (TCM) patent prescription, comprised of 4 herbs, including *H.Lév. Hutch*, is reported to be an available prescription in treating RA with fewer side effects as compares to Tripterygium tablets. To reveal the pharmacological mechanism of KX in RA treatment and side effect alleviation, we collected related information of KX from open-access databases and performed various analyses. 1354 targets were identified in KX. These targets were enriched in the calcium signaling pathway, cAMP signaling pathway, cGMP-PKG signaling pathway and PI3K-AKT signaling pathway, forming biological functions, such as cofactor binding, coenzyme binding, etc. These pathways or functions mostly affect cell cycle, differentiation, and maturation of Th17 cells, macrophage, and synovial fibroblast. These targets also act on the IL-17 signaling pathway, Th17 cell differentiation signaling pathway and TNF signaling pathway, which is related to inflammation response inhibition. Next, a disease network was constructed, which indicated IMPDH2, MTHFD1 are the key genes answering for the side effects of *H.Lév. Hutch*. The side effect–related genes lead to the negative regulation of nucleic acid, which could be restored by the rest 3 herbs through some positive amino acid metabolism. In conclusion, KX is a relatively safe alternative approach in RA intervention.

## Introduction

Rheumatoid arthritis (RA) is a chronic autoimmune disease characterized by symmetry and peripheral polyarthritis. Epidemiology shows that the prevalence of RA is 0.5% to 1%, varying by geography and population (Silman and Pearson, 2002; Alamanos et al., 2006). Chronic inflammatory arthritis often leads to joint damage and disability as the disease progressed.

Currently, treatment for RA includes non-steroidal anti-inflammatory drugs, glucocorticoids, immunosuppressants, and alternative therapy (such as Chinese medicine). KunXian Capsule (KX) is a Traditional Chinese Medicine (TCM) patent prescription which is widely used in RA treatment. There are four main herbs in KX, namely *Tripterygium hypoglaucum (H.Lév.) Hutch* (Kun Ming Shan Hai Tang), *Epimedium brevicornu Maxim* (Yin Yang Huo), *Cuscuta chinensis Lam* (Tu Si Zi), and *Lycium barbarum L* (Gou Qi Zi). Previous research has demonstrated that KX can down-regulate chemokines, inhibit inflammation response and has analgesic and immunosuppressive effects of the whole-course intervention (Jia and Liu, 2016). However, the molecular mechanism still needs to be revealed. A systematic review of 528 patients in 7 RCTs showed that the total effective rate of KX combined with methotrexate group is higher than methotrexate alone group in RA treatment (Zhou et al., 2016), the advantage lies in controlling systemic inflammation and reducing the number of swollen joints and morning stiffness. As meta-analysis reported, the adverse reactions of KX, including reproductive toxicity, liver damage, renal dysfunction, leukopenia, gastrointestinal reactions, but the adverse reaction rate is significantly lower than the Tripterygium tablet (Liu et al., 2017; Cao et al., 2018). Thus, we suspect that the drug combination of herbs in KX may work on reducing side effects.

In 2008, Hopkins proposed the concept of “network pharmacology”. Network pharmacology could provide a new strategy for drug development by analyzing the intervention of drugs on disease networks (Hopkins, 2008). It is difficult to make a detailed and comprehensive study of TCM compounds due to multi-component and multi-target characterization. Network pharmacology or system biology provides new methods for the understanding of complex Chinese medicine pharmacological mechanisms (Hopkins, 2008). At present, many databases made a significant contribution to the development of systems biology, such as the Bioinformatics Analysis Tool for Molecular mechanism of TCM (BATMAN-TCM) developed by Liu (Liu et al., 2016). BATMAN-TCM was applied in the study exploring the mechanism of *Curcuma longa L* (E’Zhu) in the treatment of breast cancer (Kong et al., 2017). In this article, we try to explore the pharmacological mechanism of KX in treating RA and explain how the drugs combination of herbs works on reducing adverse reactions. At the same time, we predicted potential therapeutic targets that guide in-depth research.

## Results

### The Biological Function of KX

**Figure 1:**
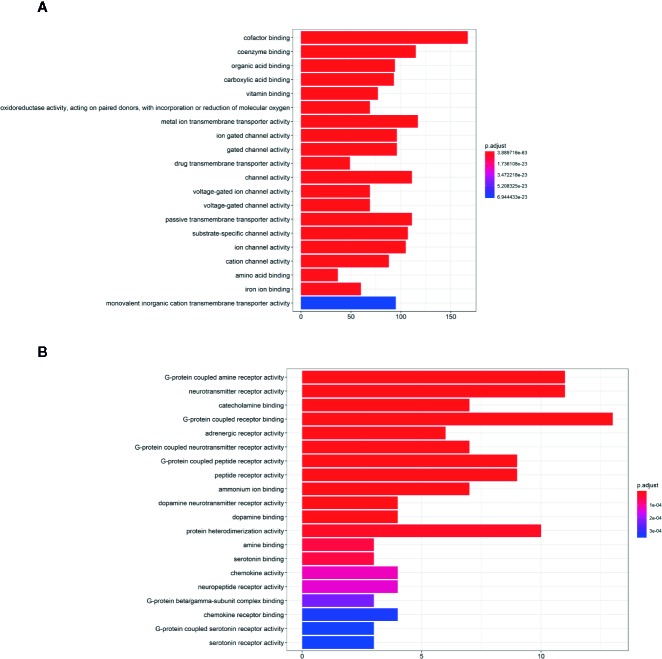
Gene ontology (GO) analysis of Kunxian Capsule. **(A)** The GO analysis of all anti-RA targets of Kunxian Capsule. **(B)** The GO analysis of hub anti-RA targets of Kunxian Capsule.

1385 targets of KX were collected from TCM-related databases, and 784 of them come from *H.Lév. Hutch*. 28635 RA-related genes were gathered from CTD. The number of overlapped targets between KX and RA is 1354, which were defined as anti-RA targets. 50 key genes were identified by CytoHubba, which were defined as hub anti-RA targets. The hub anti-RA targets included AGT, GNG2, ANXA1, etc. (**Supplementary Materials 1**). Gene Ontology (GO) analysis result showed that the main functions of anti-RA targets were: cofactor binding, coenzyme binding, organic acid binding, carboxyl binding, vitamin binding, etc. (**Figure 1A**) while the hub anti-RA targets were enriched in G protein related biological function. Such as G protein-coupled amine receptor activity, neurotransmitter receptor activity, catecholamine binding, G protein-coupled amine receptor binding, adrenergic receptor activity, G protein coupling Neurotransmitter activity, G-protein coupled peptide activity, G protein beta/gamma sub-complex complex (**Figure 1B**).

**Figure 2:**
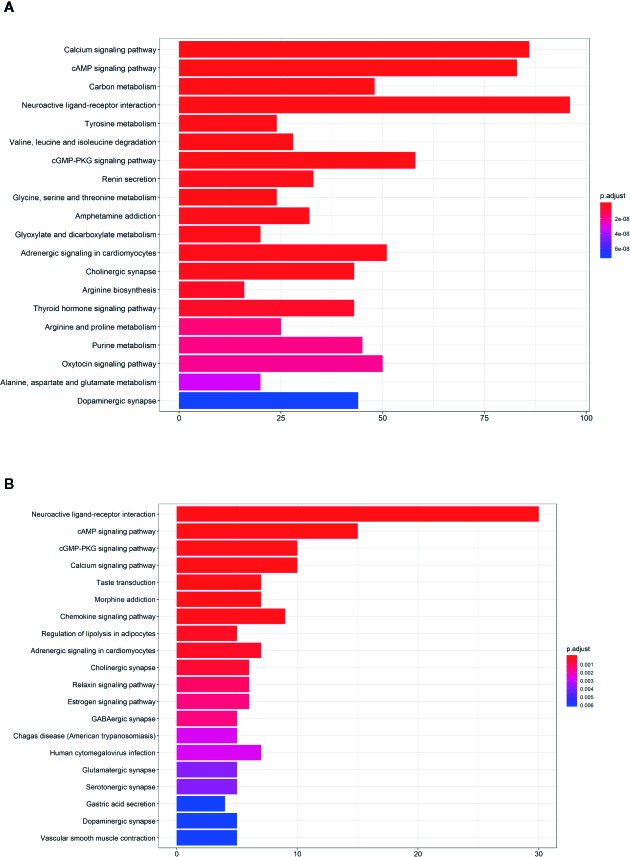
Kyoto encyclopedia of genes and genomes (KEGG) analysis of Kunxian capsule. **(A)** The KEGG analysis of all anti-RA targets of Kunxian capsule. **(B)** The KEGG analysis of hub anti-RA targets of Kunxian capsule.

We also applied KEGG pathway enrichment analysis for anti-RA targets and hub anti-RA targets. The analysis indicated that they are overlapped in the calcium signaling pathway, cAMP signaling pathway, neural ligand interaction, and cGMP-PKG signaling pathway (**Figures 2A, B**). Besides, KX is associated with various inflammatory pathways and cell cycle pathways, such as IL-17 signaling pathway, TH17 cell differentiation, TNF signaling pathway, PI3K-AKT signaling pathway. (**Supplementary Figures S1–S6**).

**Figure 3:**
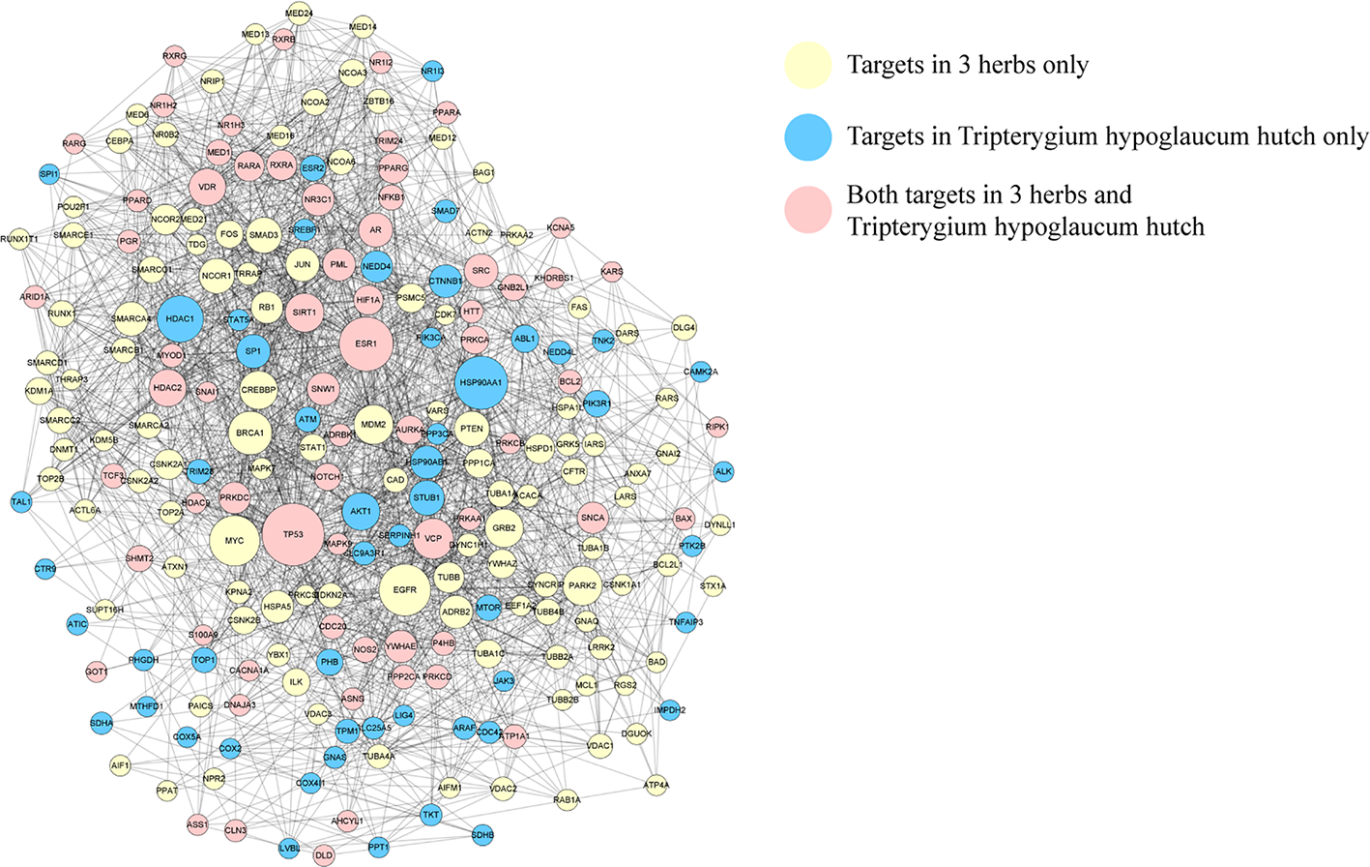
Protein protein interaction network. The yellow nodes represent targets in three herbs only, the blue nodes represent targets in *Tripterygium hypoglaucum* hutch only, the pink nodes represent the shared targets.

Protein-protein interaction (PPI) network was carried out to identify the key targets of KX in RA treatment. The whole network contains 1348 nodes, 7164 edges. For better analysis in adverse reactions and presentation, we select the nodes whose degree is above 10 to construct a PPI network (**Figure 3**).

### Mechanism of Adverse Reactions

#### Network Construction of Adverse Reactions

**Figure 4:**
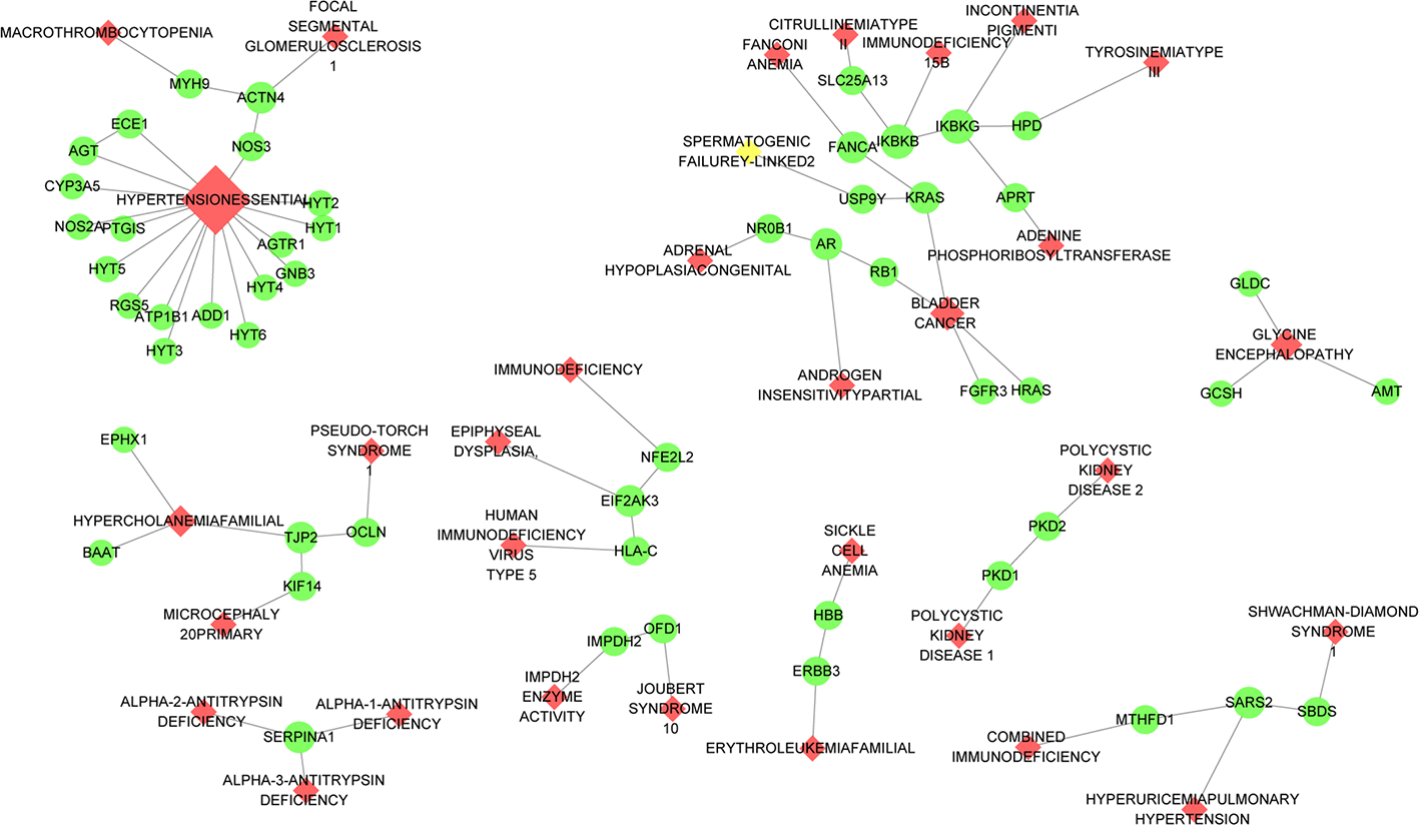
Disease network diagram. The red diamond represents some of the side effects caused by *Tripterygium hypoglaucum* hutch, and the green dots represent the disease-related genes. The direct linkage between genes was shown in PPI data of Biogrid.

From the systematic reviews mentioned above (Zhou et al., 2016), we identified 4 adverse reactions: reproductive toxicity, liver damage, renal dysfunction, leukopenia. The adverse responses involve a total of 136 genes, 9 of which were overlapped with the targets of *H.Lév. Hutch*, i.e., IMPDH2, MTHFD1, PKD2, PTGIS, SLC25A13, AGTR1, NR0B1, AR, and AGT (**Figure 4**). IMPDH2 is involved in cellular guanine metabolism, which could play a critical role in DNA and RNA synthesis (Dalal et al., 2009). MTHFD1 could encode a protein that possesses 5,10-methenyltetrahydrofolate cyclohydrolase, 5,10-methylenetetrahydrofolate dehydrogenase, and 10-formyltetrahydrofolate synthetase activities. Each of these enzymatic activities catalyzes one of three sequential reactions in the transformation of 1-carbon derivatives of tetrahydrofolate (THF), which are substrates for *de novo* purine syntheses (Pietrzik et al., 2010; Scaglione and Panzavolta, 2014). To summarize, MTHFD1 and IMPDH2 are involved in the transcriptional synthesis of RNA and DNA. Indeed, IMPDH2 is the target of Mycophenolate mofetil (MMF) (Dalal et al., 2009), and the MTHFD1, similar to methotrexate (MTX), is associated with the metabolism of THF (de Jonge et al., 2005). It is not surprising that all the side effect, such as sperm reduction and leukopenia of MMF and MTX, overlap KX’s because they shared the same mechanism. Here we consider they are instead side effects than disease treating targets because they did not enrich in main therapeutic mechanism pathways in KX.

We then analyze the rest herbs (*Epimedium brevicornu Maxim*, *Cuscuta chinensis Lam*, and *Lycium barbarum L*) of KX, which pointed out that they may help in reducing the side effect of *H.Lév. Hutch*. The analysis of the GO enrichment of rest herbs shows that the primary function involved in animal organ development, positive regulation of the cellular biosynthetic process, positive regulation of protein metabolic process, positive regulation of gene expression, etc. (**Table 1**). The KEGG enrichment analysis of the rest herbs shows that they have a regulatory effect on amino acid metabolism pathways (**Table 2**) and amino acids can be transformed into nucleic acid *via* specific metabolic pathways. To sum up, the rest herbs produce a positive regulation of the nucleic acid and amino acid biosynthetic process, positive regulation of transcription effects, resulting in consequences of alleviating adverse reactions caused by *H.Lév. Hutch*.

**Table 1:** GO analysis of the rest of 3 herbs.

Term	Count	%	P-Value	Benjamini
Animal organ development	327	33.1	7.2E-28	8.4E-26
Positive regulation of cellular biosynthetic process	215	21.8	1E-26	1.1E-24
Positive regulation of macromolecule metabolic process	299	30.3	3.7E-25	3.3E-23
Positive regulation of protein metabolic process	181	18.3	1.5E-20	8.4E-19
Positive regulation of nucleobase-containing compound metabolic process	193	19.6	1.9E-20	9.9E-19
Positive regulation of gene expression	191	19.4	9E-19	3.8E-17
Cell development	208	21.1	2.9E-17	1.1E-15
Positive regulation of transcription, DNA-templated	156	15.8	5.7E-16	1.9E-14
Positive regulation of RNA biosynthetic process	157	15.9	1E-15	3.3E-14
Positive regulation of RNA metabolic process	159	16.1	3.9E-15	1.2E-13
Cellular protein metabolic process	370	37.5	0.000000061	0.0000008
Regulation of gene expression	300	30.4	0.0025	0.015

**Table 2:** KEGG analysis of the rest of three herbs.

Term	Count	%	P-Value	Benjamini
Glycine, serine, and threonine metabolism	22	2.2	1E-11	5.8E-10
Arginine biosynthesis	16	1.6	9.9E-12	7E-10
Alanine, aspartate, and glutamate metabolism	20	2	8.4E-11	3.4E-09
Tyrosine metabolism	19	1.9	8.6E-10	0.00000003
Arginine and proline metabolism	22	2.2	3.8E-09	0.00000011
Biosynthesis of amino acids	23	2.3	0.0000012	0.000016
Tryptophan metabolism	15	1.5	0.00002	0.0002
Cysteine and methionine metabolism	13	1.3	0.00024	0.0016
Aminoacyl-tRNA biosynthesis	16	1.6	0.002	0.012
Phenylalanine, tyrosine, and tryptophan biosynthesis	4	0.4	0.0088	0.039
Drug metabolism—cytochrome P450	12	1.2	0.078	0.2

## Validation From Published Biological Evaluation

### KX Induces Apoptosis Through PI3K/AKT/mTOR Signaling Pathway

The PI3K/AKT/mTOR pathway has a pivotal role in mediating many cellular responses, including protection from apoptosis. *H.Lév. Hutch* is one of the ingredients of KX, which belongs to the *Tripterygium wilfordii* genus. Active ingredients include Tripterine (TP), Celastrol (Cel), wilforgine, etc. We made a review of the *Tripterygium wilfordii* genus herbs or their active ingredients to validate our hypothesis. The KX capsule or its active ingredients could promote varieties of cell apoptosis, including T cells, macrophages, dendritic cells, and fibroblast-like synoviocytes (FLS). Experimental evidences of KX capsule or its active ingredients for apoptosis were summarized in **Table 3**.

**Table 3:** Experimental evidence of KX capsule or its active ingredients for apoptosis.

Cell type	Ingredient	Model	Molecule mechanism	Cell phenotype	Disease phenotype	Ref
T cell	TP	TNF-Tg mice	NA	Promoted T cell apoptosis	Ameliorate	(Wang et al., 2018)
OCP	TP	TNF-Tg mice	NA	Promoted OCP apoptosis. Inhibited bone resorption. Inhibited inflammatory cytokines of macrophages	Ameliorate	(Wang et al., 2018)
Macrophage	TP	Normal macrophage	NA	Promoted macrophage apoptosis	NA	(Bao et al., 2006)
DC	TP	LPS-induced DC	Inhibit the activation of NF-κB, p38 MAP kinase, and Caspase 3	Promote DC apoptosis and inhibit inflammatory cytokines of DC	NA	(Liu, 2004)
FLS and SF	Cel	AIA rats and SF of RA patients	Ca2+/calmodulin-dependent kinase kinase-β-AMP-activated protein kinase-mTOR pathway	Triggered Ca2+ signaling to induce autophagic cell death in FLS/SF.	Ameliorate	(Wong et al., 2019)
Granulosa cell	TP	Normal granulosa cells in rats	Decreased the protein levels of TGF-1, Smad2, AKT, and CCND2	Inhibited cell proliferation in rat granulosa cells	Promote side effects	(Su et al., 2014)

TNF, tumor necrosis factor; OCP, osteoclast precursors; DC, dendritic cell; LPS, lipopolysaccharide; NF-κB, nuclear factor kappa-B; FLS, fibroblast-like synoviocytes; SF, synovial fibroblasts; AIA, adjuvant-induced arthritis; AMP, adenosine monophosphate; mTOR, mammalian target of rapamycin; TGF, transforming growth factor; SMAD, suppressor of mothers against decapentaplegic; AKT, protein kinase B (PKB), also known as AKT; CCND2, cyclin D2.

### The KX Capsule or Its Ingredients Inhibit the Inflammatory Network of Th17, Macrophage, and FLS

The KX capsule or its ingredients could target at NF-κB, AP-1, IKK, HIF-1α, resulting in varieties of phenotype changes, such as inhibition of inflammatory cytokines released by macrophages, differentiation of T cell, invasion of FLS. Experimental evidence of the KX capsule or its active ingredients for inhibiting inflammatory network were summarized in **Table 4**.

**Table 4:** KX capsule or its active ingredients for inhibiting inflammatory network.

Cell type	Ingredient	Model	Molecule mechanism	Cell phenotype	Disease phenotype	Ref
Th17	Cel	E. coli-induced THP-1 macrophage-like cell line and AIA Wistar rats	Inhibits the activation of NF-kB and caspase-1	Prevented Th17 infiltration	Ameliorate	(Cascão et al., 2012)
Macrophage	Cel	E. coli-induced THP-1 macrophage-like cell line and AIA Wistar rats	Inhibits the activation of NF-kB and caspase-1	decreases IL-1β and TNF secreted by macrophages	Ameliorate	(Cascão et al., 2012)
T cell	PG27	T cell	Inhibited activity of IKK kinase, AP-1, and MAPK	Inhibited T cell activity	NA	(Ho et al., 2013)
T cell	TP	T cell	Inhibited activity of IKKα, AP-1, IKKβ, and MAPK	Inhibited T cell activity	NA	(Ho et al., 2013)
Macrophage	*Tripterygium wilfordii* polycoride	LPS-induced macrophages	Inhibited the expression of TLR4 and NF-κB p65-	Regulated inflammatory cytokines in macrophages	NA	(Ping et al., 2015)
Macrophage	*Tripterygium wilfordii* polycoride	CFA Wistar rats and LPS-induced RAW 264.7 macrophages	NA	Decreased cytokine IL-1β, IL-6, and TNF-α produced by macrophages	Ameliorate	(Tong et al., 2018)
FLS	Cel	Hypoxia-induced FLS	Suppressed the binding activity of HIF-1α in the CXCR4 promoter, and blocked hypoxia-induced accumulation of nuclear HIF-1α.	Suppressed hypoxia-induced FLS migration and invasion.	NA	(Li et al., 2013)

Cel, celastrol; NF-κB, nuclear factor kappa-B; AIA, adjuvant-induced arthritis; IL, interleukin; TNF, tumor necrosis factor; IKK, IκB kinase; AP-1, activator protein 1; MAPK, mitogen-activated protein kinase; LPS, lipopolysaccharide; TLR, Toll-like receptors; FLS, fibroblast-like synoviocytes.

### The Relationship Between Amino Acid Metabolism and Toxicity

Nucleotide synthesis, a biological process that needs the exitance of cofactor tetrahydrofolate, is required in cell survival. Methotrexate (MTX), the main treatment in RA, inhibits the enzyme dihydrofolate reductase and then lead to tetrahydrofolate exhausted. The depletion of tetrahydrofolate causes cell death by suppressing DNA and RNA production (Kanarek et al., 2018). Toxicants, such as deoxynivalenol, could cause amino acids and nucleotides deficiency through repressing a variety of biosynthesis of amino acids and nucleotides, finally lead to hepatotoxicity, immunotoxicity and other toxicity (Ji et al., 2016; Geng et al., 2019; Tang et al., 2019). Furthermore, disruption of the TCA cycle and glutamate metabolism plays an essential role in the pathogenesis of amyotrophic lateral sclerosis (ALS), a disease that is closely related to the death of the neurocytes (Veyrat-Durebex et al., 2016). For cancer cell proliferation, these cells up-regulate serine synthesis, one-carbon (folate) metabolism, and the glycine cleavage system (SOG pathway) to fulfill the biosynthetic requirements of purines, ATP and NADPH. By contrast, MTX could inhibit the proliferation of cancer cells *via* disrupting the pathways (Tedeschi et al., 2013). TP (one of the active ingredients of *H.Lév. Hutch*) increases the level of cysteinylglycine and cysteine conjugates derived from NADPH-independent metabolism and promotes hepatotoxicity, which could be ameliorated by the supplement of glutathione (Du et al., 2014). Another active ingredient of *H.Lév. Hutch*, Cel suppresses tryptophan catabolism, shows its cytotoxic effect against colon cancer cells (Qi et al., 2018).

In contrast, positive regulation of the amino acid metabolism or supplement of some amino acids may help in cell survival and detoxication. MTX induces an increase in intestinal permeability. After MTX treatment, the Caco-2 cells barrier function is decreased, which was assessed by transepithelial electrical resistance, but could be reversed by the supplement of glutamine, glutamate, arginine, and leucine (Beutheu et al., 2013). Similarly, MTX-induced gastrointestinal toxicity in rats could be alleviated by exogenous glutamine and arginine (Gulgun et al., 2010) and supplement of branched-chain amino acids are beneficial effects on neuronal survival and axon regeneration (Park et al., 2017). *Epimedium brevicornu Maxim*, *Cuscuta chinensis Lam*, and *Lycium barbarum L* play a pivotal role in amino acid or nucleotide metabolism. *Lycium barbarum L* contains a variety of amino acids. Up to now, 18 amino acids have been identified, including 8 essential amino acids. This is the only plant that has been reported to contain taurine (Luo et al., 2004; Tian and Wang, 2006; Hsieh et al., 2018). Supplementation with *Lycium barbarum* polysaccharides (LBP) stimulates the growth and metabolic activity *via* increasing amino acid and nucleotide metabolism, short-chain fatty acid-related metabolism (Wang et al., 2019b). Significant metabolic disorders were observed in rats with a prolonged injection of hydrocortisone. Epimedium rebalances the multiple metabolic pathways involved in amino acid and phospholipid metabolism, for example, up-regulate taurine, glycine, and β-glucose levels (Huang et al., 2013; Pan et al., 2016). By far, there is insufficient data to reveal the three herbs in ameliorating *H.Lév. Hutch*-related side effects *via* exact molecule mechanism or amino acid/nucleotide metabolism. Indeed, the three herbs alleviate toxicity in different ways. Total flavonoids of Epimedium protect the reproductive system against cyclophosphamide-induced toxicity by inhibiting oxidative stress (Yuan et al., 2014). By contrast, *Cuscuta chinensis Lam* detoxicates cyclophosphamide-induced toxicity through regulating cytokines GM-CSF and TNF-α (Raju et al., 2015). Herein, for endeavoring to illustrate the coexisting mechanism of causing and alleviating side effects, we supposed amino acid and nucleotide metabolism approach.

## Discussion

**Figure 5:**
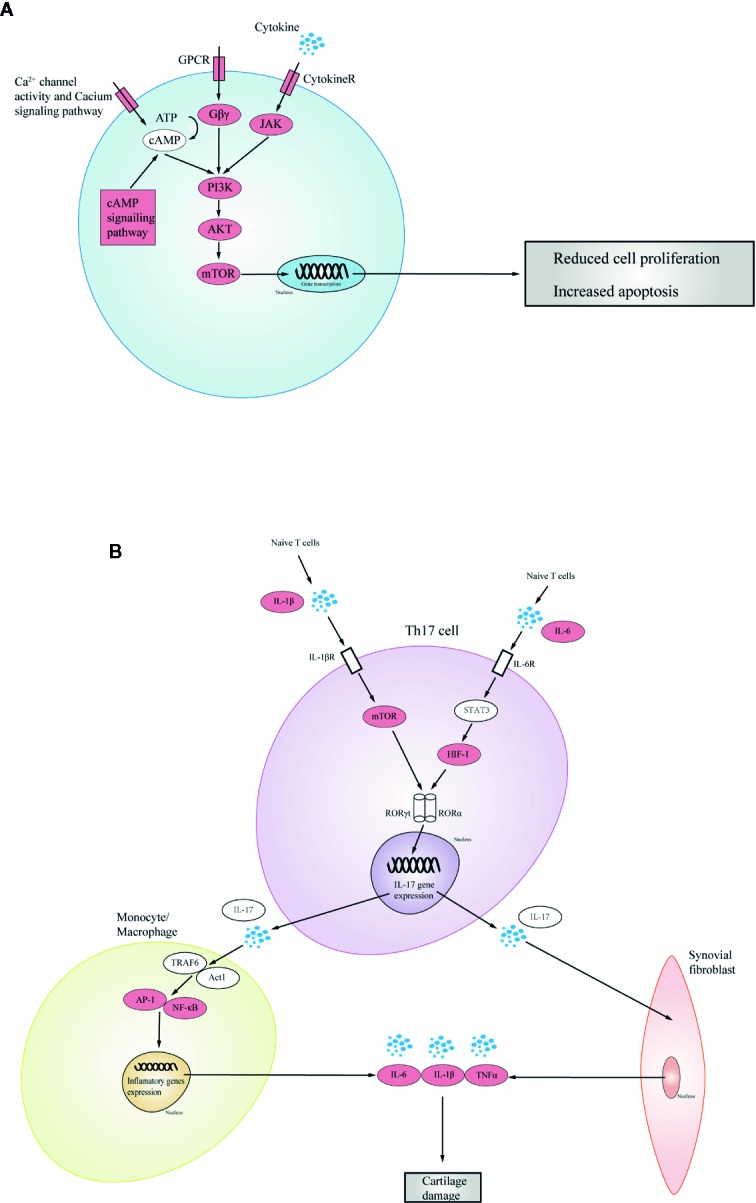
Mechanism of Kunxian capsule in treating RA. The red nodes represent targets in Kunxian capsule. **(A)** Through intermediate protein Gβγ, GPCRs regulated the activation of PI3Ks. The phosphorylation of PI3K activated AKT which lead to downstream cascades, such as controlling cell proliferation, protein translation. JAK-STAT signaling is related with the proliferation of the inflammatory cells. Lastly, cAMP, a second messenger affected by calcium signaling pathway, could activate PI3K via intermediate molecules, such as PKA and mTOR, which are high degree targets in KX. KX inhibits the three pathways and decrease cell proliferation and promote cell apoptosis in RA. **(B)** Th17 cells could be activated by IL-1β (through mTOR-ROR) or IL-6 (through STAT3-HIF1-ROR). Subsequently, the activated TH17 cells released IL-17, which activated the downstream effector cells, such as monocytes, macrophages, and synovial fibroblast. These cells could release inflammatory cytokines which could promoted cartilage damage. KX could target at the critical checkpoints in the pathways, downregulating the inflammatory cells proliferation and alleviating cartilage damage.

KX can act on the calcium signaling pathway, cAMP signaling pathway, cGMP-PKG signaling pathway, and G protein-coupled receptors (GPCRs). All these pathways are involved in cell signal transmission and could have crosstalk with PI3K/AKT/mTOR signaling pathway, which is used to achieve the proliferation, differentiation, and apoptosis of immune cells in RA (Shu et al., 2017; Feng and Qiu, 2018; Wang et al., 2019a). In physiological states, regulation of PI3K by GPCRs mainly occurs through Gβγ, which directly binds to the p110 catalytic subunit and protein kinase A (PKA) (Wang et al., 2019a). PI3K phosphorylates AKT, which has many downstream cascades, including mTOR, a transcription factor encodes genes of cell proliferation and survival (Keppler-Noreuil et al., 2016). Second, JAK-STAT signaling can interconnect with the PI3K/AKT/mTOR pathway (Rawlings et al., 2004). Given that JAKs are associated with cytokine receptors, such as cytokines IFNγ, IL-2, IL-4, and IL-10 (Rodig et al., 1998), it may exist a signaling transmission from cytokines receptors to mTOR. Lastly, cAMP, a second messenger, could be affected by the calcium signaling pathway, could activate PI3K *via* intermediate molecule such as PKA. The molecules mentioned above were high degree targets in KX, once they were inhibited, resulting in the decrease of proliferation, differentiation, and apoptosis of immune cells in RA (**Figure 5A**).

At the same time, KX can alleviate the inflammatory response and reduce the disease activities by regulating multiple key inflammatory signaling pathways in RA, such as IL-17 signaling pathway, Th17 cell differentiation signaling pathway, and TNF signaling pathway (Lubberts, 2015). In physiological status, Th17 cells could be activated by IL-1β (through mTOR-ROR) or IL-6 (through STAT3-HIF1-ROR). Subsequently, IL-17 released by Th17 stimulated the downstream effector cells, such as monocytes, macrophages, and synovial fibroblasts. These cells could release inflammatory cytokines, which could promote cartilage damage. KX could target at the critical checkpoints in the pathways, downregulating the inflammatory cells proliferation and alleviating cartilage damage (**Figure 5B**).

In terms of adverse reactions, it was found that *H.Lév. Hutch* can act on IMPDH2 and MTHFD1.IMPDH2 encodes the rate-limiting enzyme (inosine 5′-monophosphate dehydrogenase) in the *de novo* guanine nucleotide biosynthesis. It is thus involved in maintaining cellular guanine deoxy- and ribonucleotide pools, which needed for DNA and RNA synthesis (Dalal et al., 2009). The functions of MTHFD1 have been described previously. Tetrahydrofolate is an essential coenzyme in the one-carbon transferase system. The coenzyme is produced by folic acid catalyzed by folate reductase to form dihydrofolate. Dihydrofolate is then catalyzed by dihydrofolate reductase to form tetrahydrofolate. Tetrahydrofolic acid is a carrier of the one-carbon group, which can participate in the purine’s synthesis, and promote the formation of healthy blood cells (Watkins et al., 2011).

**Figure 6:**
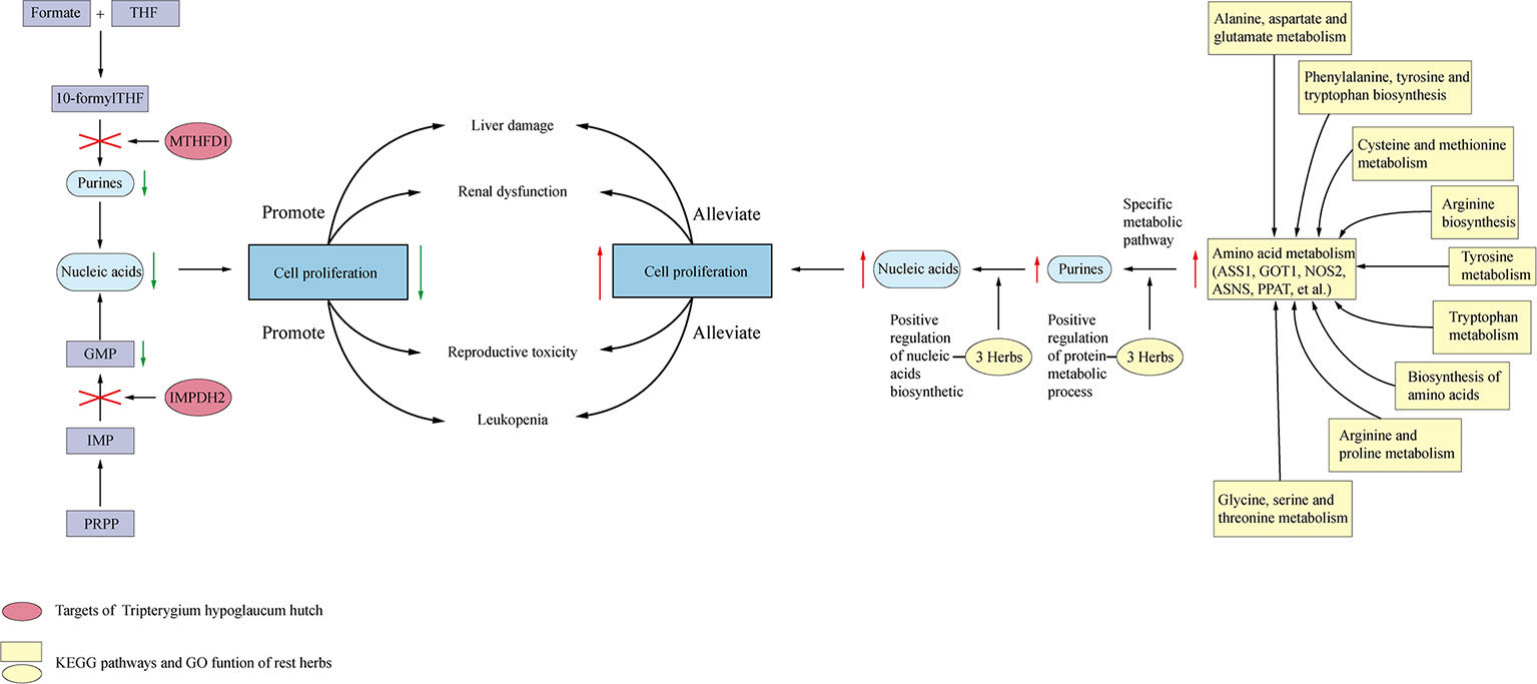
The mechanism of side effect caused by *Tripterygium hypoglaucum* hutch and the mechanism of alleviation compensated by the rest of herbs. *Tripterygium hypoglaucum* hutch may inhibit the activity of IMPDH2 which encodes the rate-limiting enzyme (inosine 5′-monophosphate dehydrogenase) in the *de novo* guanine nucleotide biosynthesis. As a result, the metabolism of nucleic acid was inhibited because of the downregulation of guanine. Besides, *Tripterygium hypoglaucum* hutch may inhibit the activity of MTHFD1 which encodes a protein that possesses the folic acid metabolism related enzymes activities, disrupting the purines converted to nucleic acid. The rest herbs could act on ASS1, GOT1, NOS2 et al. in the amino acid metabolism pathways, including glycine, serine, and threonine and form a positive regulation of amino acid. After that amino acid could be changed to purines *via* specific metabolic pathway, which compensating purines metabolic disrupted by *Tripterygium hypoglaucum* hutch. Besides, the rest herbs could play roles in positive regulation of gene expression, positive regulation of transcription, DNA-templated, positive regulation of RNA biosynthetic process, helping in the transition of purines to nucleic acid.

IMPDH2 and MTHFD1 are involved in nucleic acid metabolism and are responsible for perm reduction and leukopenia. Targets of ASS1, GOT1, NOS2 in rest herbs form a positive regulation of amino acid. Subsequently, amino acid could be changed to purines *via* the specific metabolic pathway, which compensating purines metabolic disrupted by *H.Lév. Hutch*. Besides, the rest herbs take effect in the transition of purines to nucleic acids (**Figure 6**). Interestingly, they enriched in drug metabolism-cytochrome P450 signaling pathway, indicating that they may help in promoting drug metabolism.

Zhang (Zhang et al., 2014) has validated that *H.Lév. Hutch*, one herb in KX, could decrease the IL-17 level in serum in the CIA rats, but the exact mechanism remains unknown. Although some targets have been validated by different researchers using active ingredients, there may be synergism, antagonism, potentiation to show us a different result. Some of the targets with high degrees on these two pathways, such as mTOR, NF-κB, can be experimentally verified in the future. We speculate that KX can inhibit the targets mentioned above, decreasing TH17 cell differentiation and reducing IL-17 levels. It also needs to verify that whether KX could disrupt the PI3K-AKT-mTOR pathway and, thus, promote the apoptosis of related inflammatory cells. Interestingly, KEGG enrichment analysis showed that KX also has neuroactive ligand-receptor interaction, which may have central analgesic effects.

The mechanism prediction of KX *via* bioinformatics is limited because the minority components of the herbs are analyzed in our study, which may make little sense in clinical practice. However, we believe that the systematic approach to infer therapeutic mechanisms of KX for RA treatment provides new insights into the understanding of this TCM formula and provides a new perspective in TCM patent prescription studies. What is more, we provide new insights into the understanding of drug matching of TCM to alleviate side effects.

## Conclusion

KX treats RA *via* inhibiting the inflammatory network and inducing apoptosis through PI3K/AKT/mTOR signaling pathway. *H.Lév. Hutch*, one of the ingredients of KX, could cause some side effects, such as sperm reduction and leukopenia. The rest 3 herbs (*Epimedium brevicornu Maxim*, *Cuscuta chinensis Lam*, and *Lycium barbarum L*) could play a role of detoxication through positive regulation of the amino acid metabolism or supplement of some amino acids. In conclusion, KX is a relatively safe alternative approach in RA treatment.

## Materials and Methods

### Target Prediction of Kunxian Capsule

There are four main components of KX, *H.Lév. Hutch* (Kun Ming Shan Hai Tang), *Epimedium brevicornu Maxim* (Yin Yang Huo), *Cuscuta chinensis Lam* (Tu Si Zi), and *Lycium barbarum L* (Gou Qi Zi). The component and targets of *Epimedium brevicornu Maxim* (Yin Yang Huo), *Cuscuta chinensis Lam* (Tu Si Zi), and *Lycium barbarum L* (Gou Qi Zi) were searched at BATMAN-TCM. The Cut-off value is set to 20. Since the BATMAN-TCM (http://bionet.ncpsb.org/batman-tcm/) database does not record *H.Lév. Hutch* component data, a literature search is required. Finally, we used the data of a published master’s thesis in Chinese (Li, 2019). The thesis summarized the components of *H.Lév. Hutch*, which has been identified by now. Then the compound was sorted and converted to PubChem_CID in PubChem (https://pubchem.ncbi.nlm.nih.gov/).

### Screening of Related Genes and Targets

CTD is a publicly available database that contains robust disease genes. “Rheumatoid arthritis” was entered into the CTD database to obtain any possible disease target genes.

OMIM is a comprehensive, authoritative compendium of human genes and genetic phenotypes database, which can be used to establish disease networks *via* pathological disorder. The OMIM database was retrieved by PubMed, and the retrieved relevant disease genes were recorded.

### Visualization and Functional Analysis

#### Drug-Target-Disease Interaction Network and Adverse Reaction Disease Network Construction

Targets of the Kunxian capsule and RA-related genes were input into STRING (https://string-db.org/) to attain protein interaction networks, with a minimum required interaction score 0.9. Cytoscape 3.7.0 (https://cytoscape.org/) software was applied for PPI visualization. The adverse reaction disease network was constructed using the gene retrieved in OMIM with a minimum required interaction score of 0.4.

##### Screening of Hub Genes

CytoHubba, a novel Cytoscape plugin that can be used to rank node features in the network. CytoHubba provides 11 topological analysis methods, including Degree, Edge Percolated Component, Maximum Neighborhood Component, Density of Maximum Neighborhood Component, Maximal Clique Centrality, and six shortest path centralities (Bottleneck, EcCentricity, Closeness, Radiality, Betweenness, Stress). Among the 11 methods, the MCC PPI network has better performance in predicting the accuracy of key targets (Shu et al., 2017). The MCC algorithm is used to construct the PPI network and select the top 50 key targets.

#### GO Enrichment and KEGG Pathway Analysis

Bioconductor’s “clusterProfiler,” “pathview,” and other functional packages (Hueber et al., 2010; Li et al., 2012; Jiao et al., 2018) in the R software were utilized for GO enrichment, KEGG pathway analysis, and plotting. The cutoff values and P value are both set to 0.05, and the top 20 results are selected for drawing.

## Data Availability Statement

The original contributions presented in the study are included in the article/**Supplementary Material**, further inquiries can be directed to the corresponding authors.

## Author Contributions

YT: data analyses, figure preparation, manuscript preparation, and study initiation. YZ: interpretation, data analyses, and manuscript preparation/submission. LL: data analyses, and manuscript preparation. ZX: data interpretation, figure preparation. CW: critically reviewed the manuscript, project funding. LH: critically reviewed the manuscript and study initiation. All authors approved the final version of the manuscript.

## Funding

Research was funded by National Key R&D Program of China (2018YFC1705500).

## Conflict of Interest

The authors declare that the research was conducted in the absence of any commercial or financial relationships that could be construed as a potential conflict of interest.

## Abbreviations

RA, Rheumatoid arthritis; KX, KunXian capsule; (H.Lév.) Hutch, Tripterygium hypoglaucum Hutch; TCM, Traditional Chinese medicine; BATMAN-TCM, Bioinformatics analysis tool for molecular mechanism of TCM; AGT, Angiotensinogen; GNG2, G protein subunit gamma 2; ANXA1, Annexin A1; GO, Gene ontology; TNF, Tumor necrosis factor; PI3K, Phosphoinositide 3-kinase; AKT (also known as PKB), Protein kinase B; PPI, Protein-protein interaction; IMPDH1, Inosine monophosphate dehydrogenase 1; MTHFD1, Methylenetetrahydrofolate dehydrogenase, cyclohydrolase, and formyltetrahydrofolate synthetase 1; PKD2, Polycystin 2; PTGIS, Prostaglandin I2 synthase; SLC25A13, Solute carrier family 25 member 13; AGTR1, Angiotensin II receptor type 1; NR0B1, Nuclear receptor subfamily 0 group B member 1; AR, Androgen receptor; THF, Tetrahydrofolate; MMF, Mycophenolate mofetil; MTX, Methotrexate; mTOR, Mechanistic target of rapamycin kinase; TP, Tripterine; Cel, Celastrol; FLS, Fibroblast-like synoviocytes; NF-κB, Nuclear factor kappa B; AP-1, Activator protein 1; IKK, IκB kinase; HIF-1α, Hypoxia-inducible factor-1α; TCA cycle, Tricarboxylic acid cycle; ALS, Amyotrophic lateral sclerosis; LBP, *Lycium barbarum* polysaccharides; GM-CSF, Granulocyte-macrophage colony-stimulating factor; GPCRs, G protein-coupled receptors; PKA, Protein kinase A; JAK, Janus kinase; STAT, Signal transducer and activator of transcription; IFNγ, Interferon gamma; IL, Interleukin; Th, T helper cell; ASS1, Argininosuccinate synthase 1; GOT1, Glutamic-oxaloacetic transaminase 1; NOS2, Nitric oxide synthase 2; CIA, Collagen-induced arthritis.
