# Supplementary material for: Kunxian Capsule for Rheumatoid Arthritis: Inhibition of Inflammatory Network and Reducing Adverse Reactions Through Drug Matching
Source: Front Pharmacol. 2020 Apr 17;11:485. doi: 10.3389/fphar.2020.00485 (PMC7181472; doi:10.3389/fphar.2020.00485)

Supplementary Material 1

# Supplementary Data

## Search strategy for disease network (via pubmed)

For reproductive toxicity

reproductive toxicity[Text Word]

reproduction toxicity[Text Word]

oligospermia[Text Word]

menstrual disorder[Text Word]

For leukocytopenia

leukocytopenia[Text Word]

leukopenia[Text Word]

oligoleukocythemia[Text Word]

For renal damage

renal damage[Text Word]

renal injury[Text Word]

renal impairment[Text Word]

kidey damage[Text Word]

kidey injury[Text Word]

kidey impairment[Text Word]

For liver damage

liver damage[Text Word]

liver injury[Text Word]

hepatic injury[Text Word]

# Supplementary Figures and Tables

## Supplementary Tables

Table 1. Hub anti-RA targets

| Gene name | Gene name | Gene name | Gene name | Gene name |
| --- | --- | --- | --- | --- |
| AGT | GPR18 | CCL5 | ADRA2C | GRM7 |
| GNG2 | CXCL13 | AGTR2 | GPER1 | CNR1 |
| ANXA1 | ADCY1 | SUCNR1 | CHRM2 | HTR1A |
| BDKRB2 | ADRA1A | DRD2 | SST | ADRA2A |
| DRD3 | TACR2 | PF4 | GNAI2 | OXER1 |
| CX3CR1 | TAC1 | NPY2R | OPRK1 | RXFP4 |
| CNR2 | ADRA1D | HTR1B | C5 | ADRA2B |
| CHRM4 | GNA15 | TAS1R3 | HTR1D | TAS1R1 |
| DRD4 | CXCR4 | ADCY3 | OPRM1 | HCAR2 |
| GABBR1 | PTGER3 | ADORA1 | HCAR3 | TAS1R2 |

## Supplementary Figures

### Supplementary Figure S1. CALCIUM SIGNALING PATHWAY


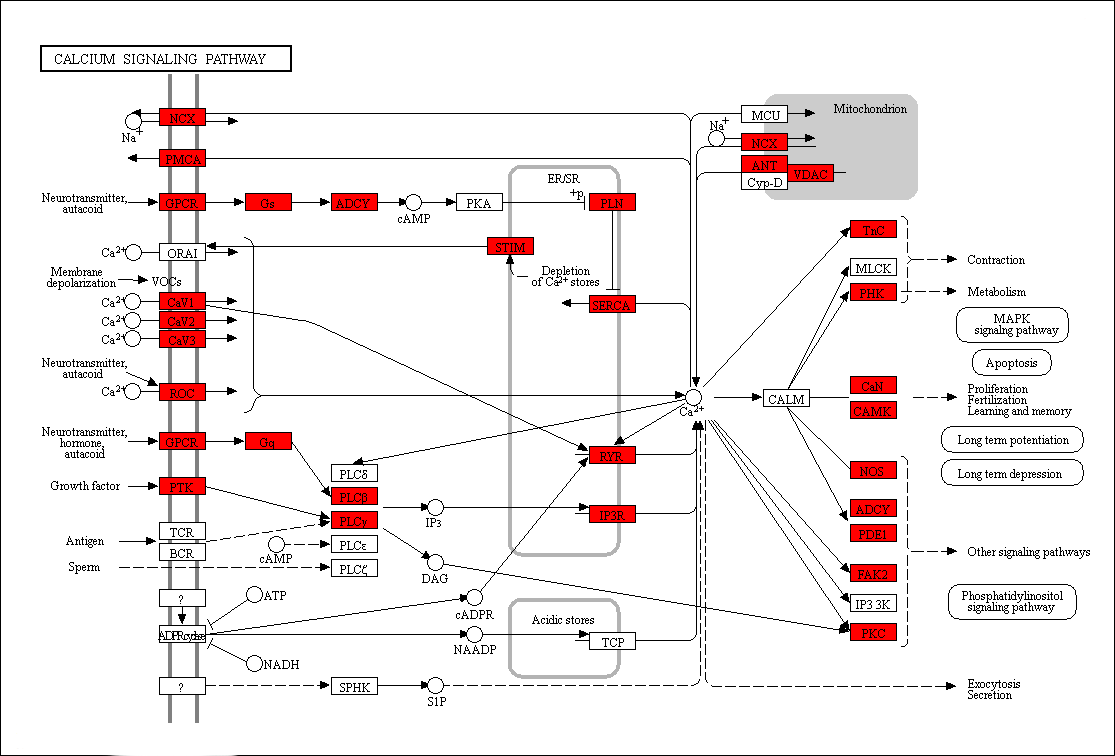


### Supplementary Figure S2. TNF SIGNALING PATHWAY


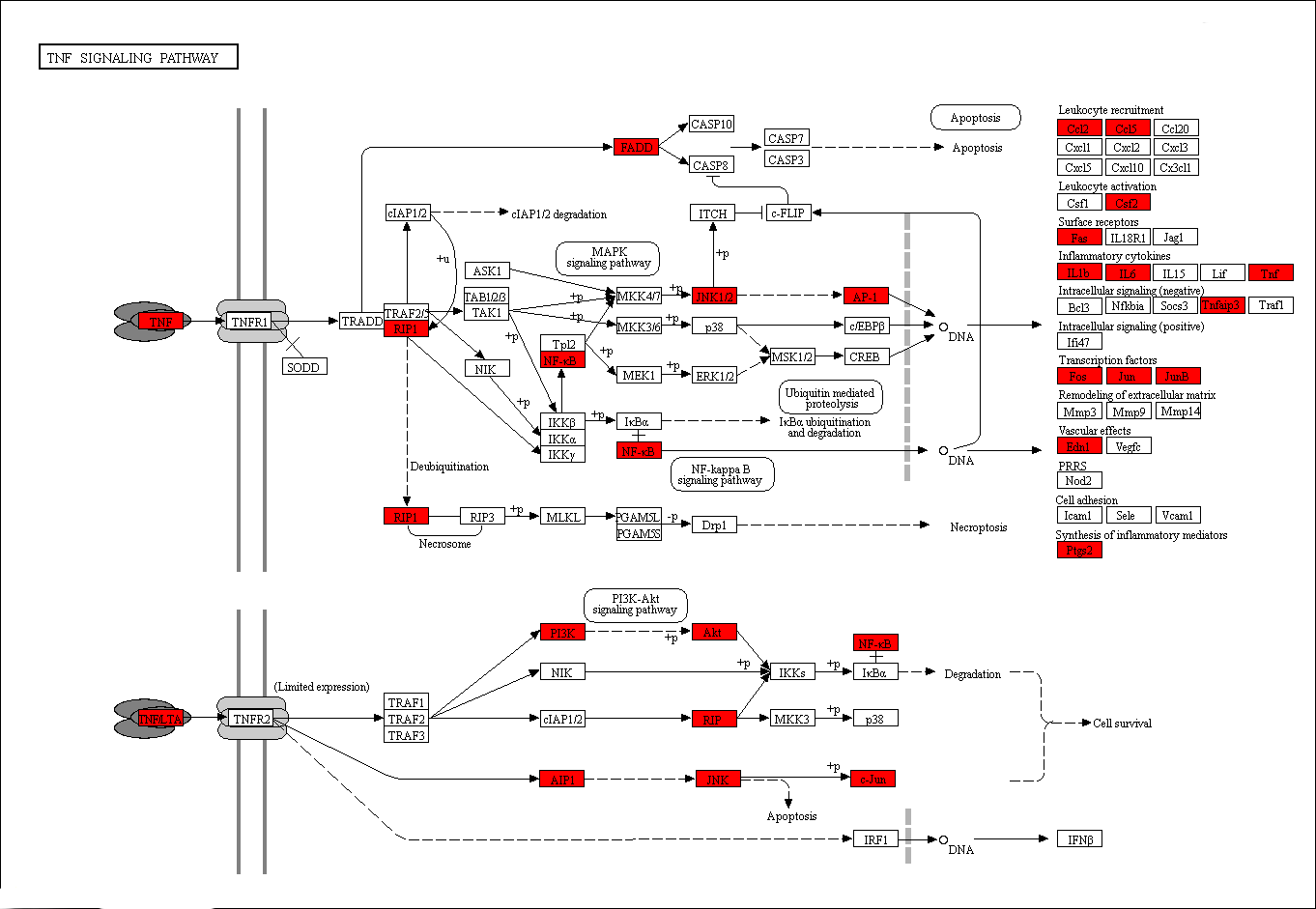


### Supplementary Figure S3. cAMP SIGNALING PATHWAY


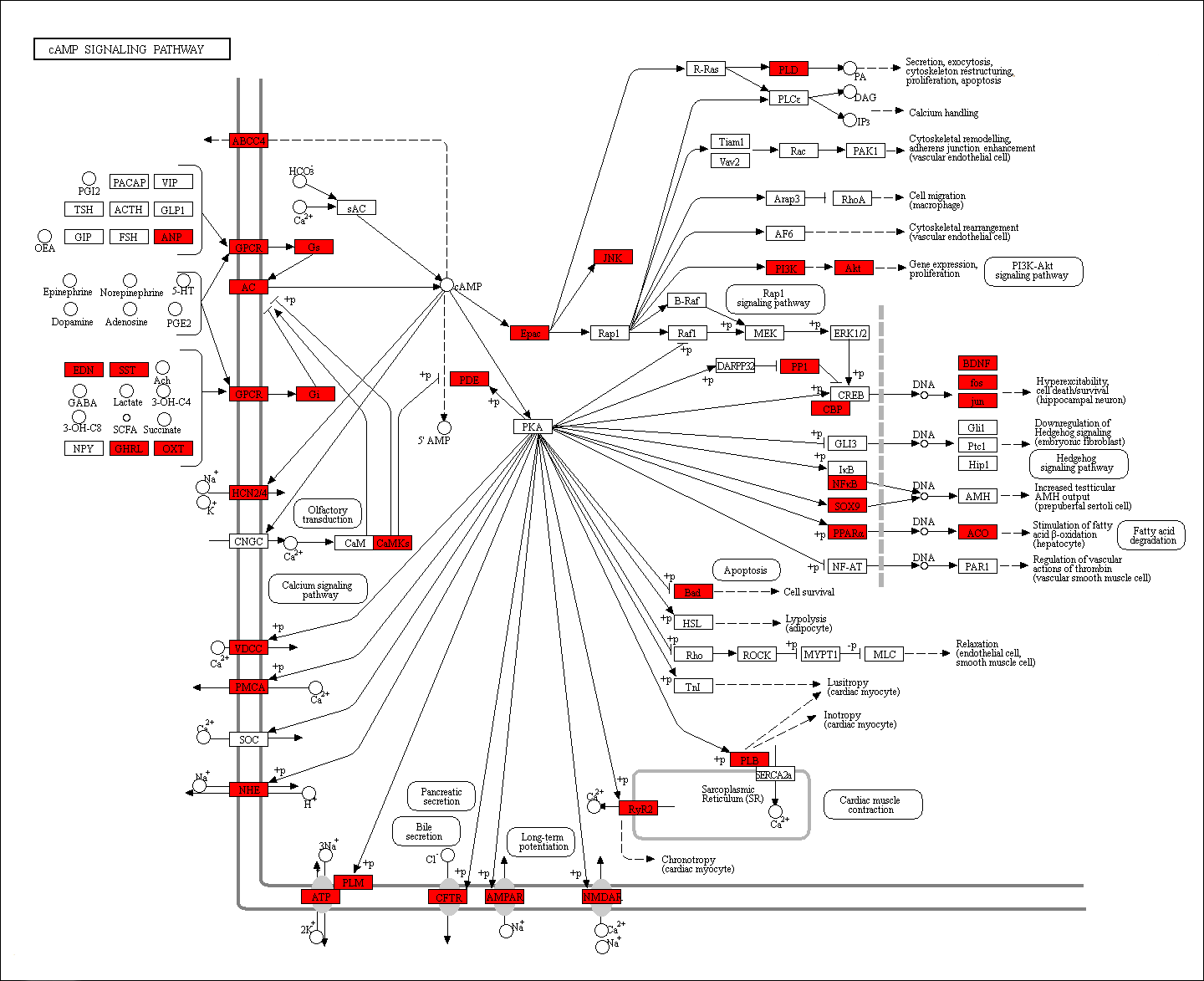


### Supplementary Figure S4. PI3K-AKT SIGNALING PATHWAY


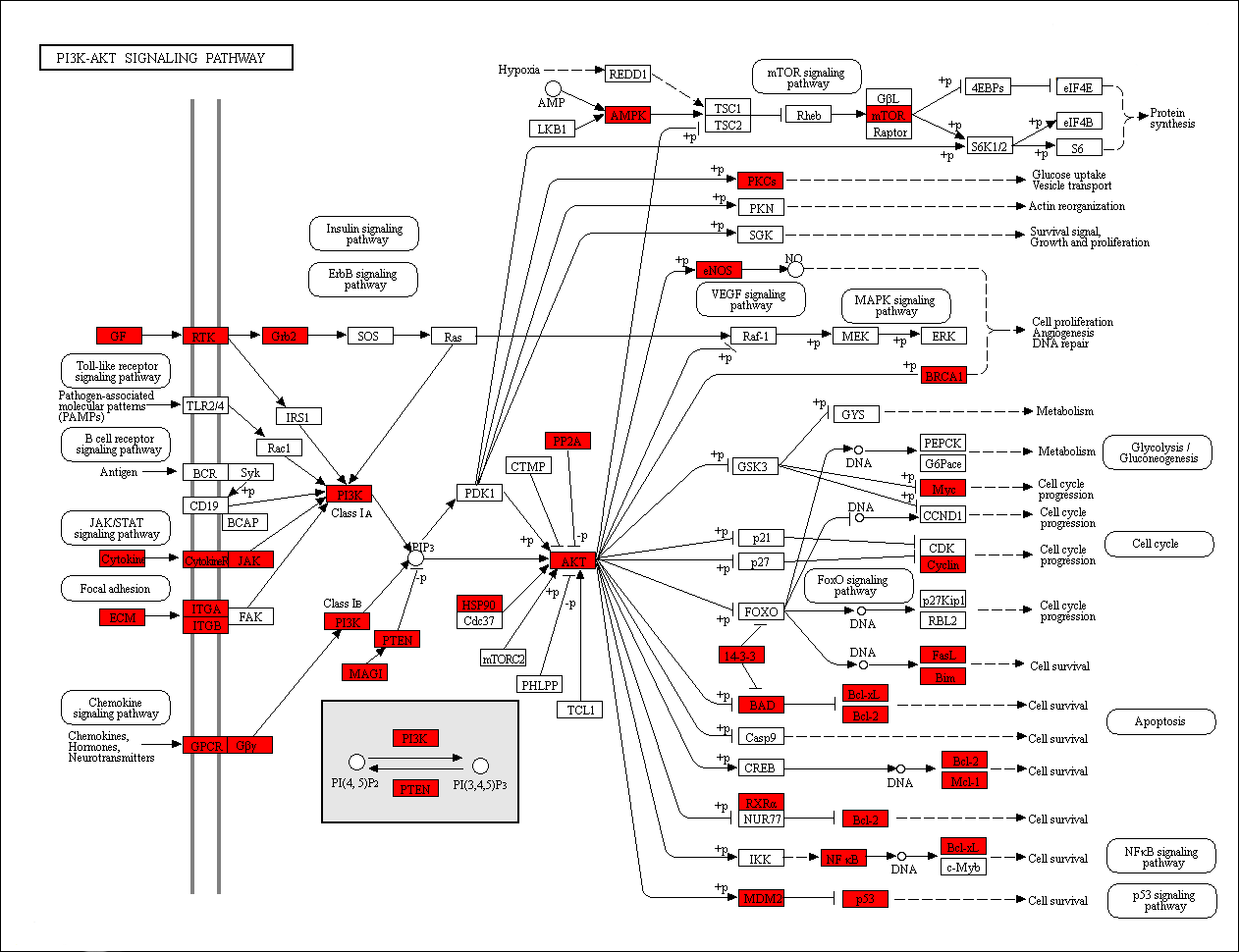


### Supplementary Figure S5. Th17 SIGNALING PATHWAY


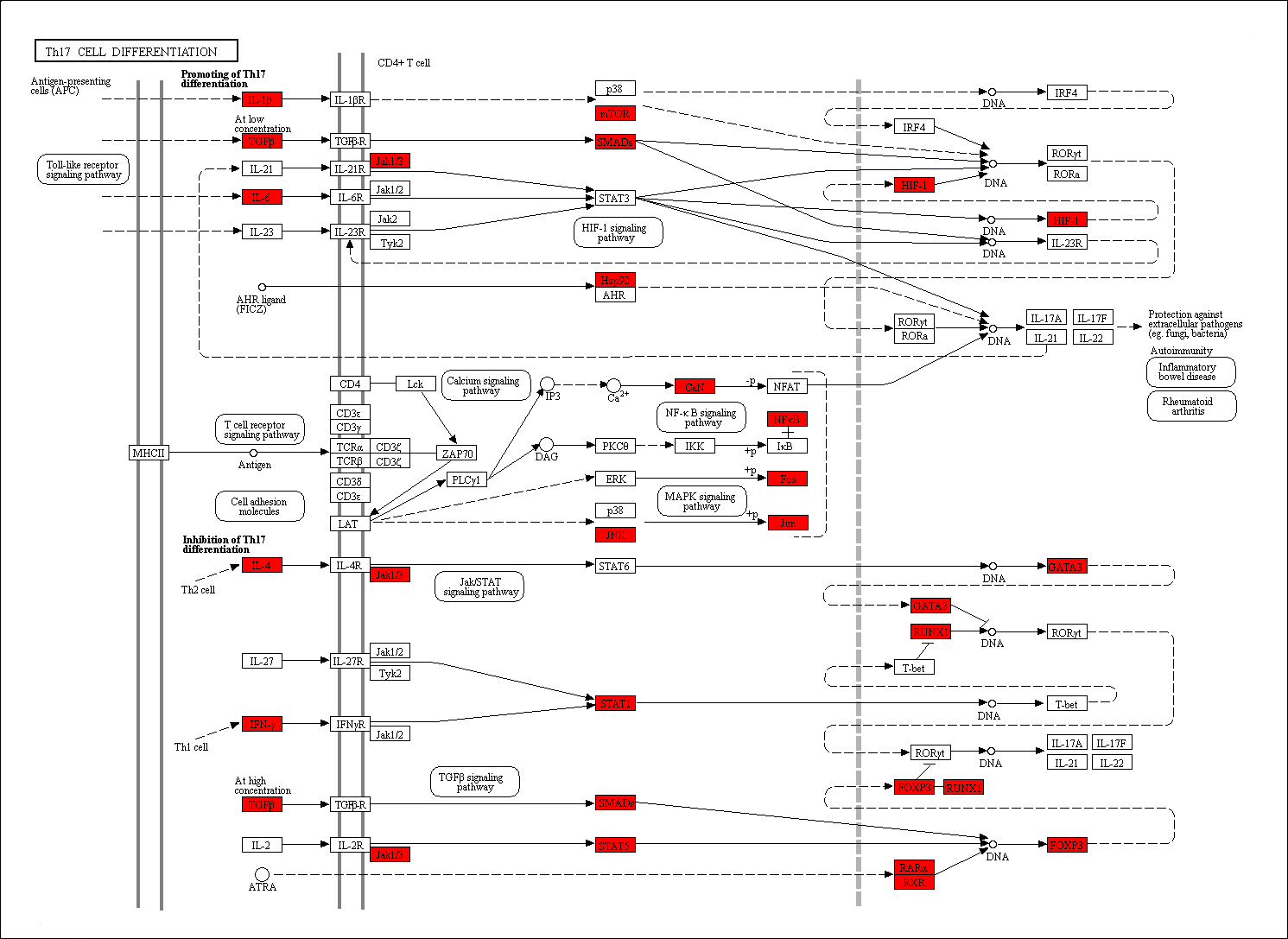


### Supplementary Figure S6. IL-17 SIGNALING PATHWAY


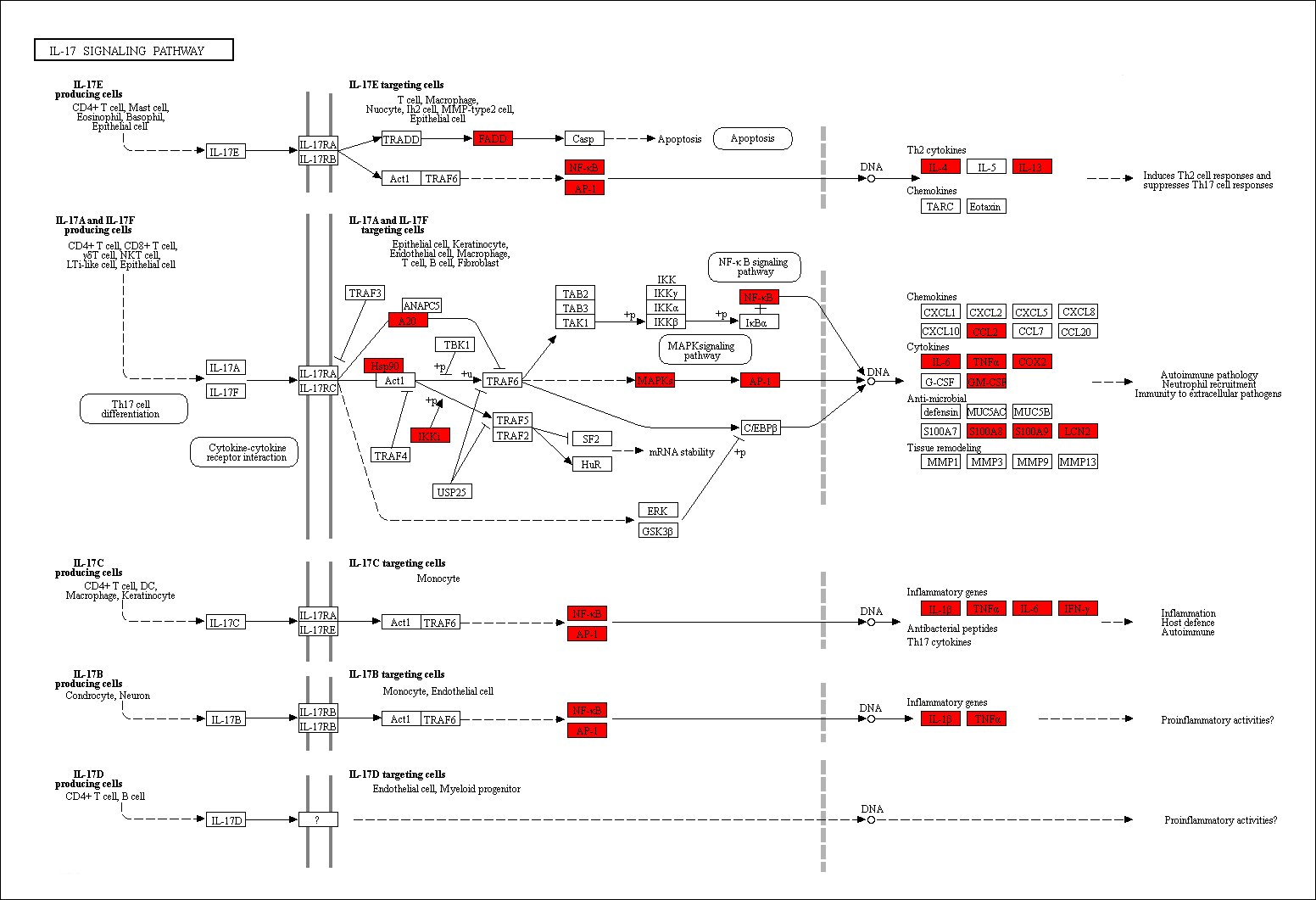

Supplement: Supplementary file 1 [file DataSheet_1.docx]
