# Supplementary material for: Kunxian Capsule for Rheumatoid Arthritis: Inhibition of Inflammatory Network and Reducing Adverse Reactions Through Drug Matching
Source: Front Pharmacol. 2020 Apr 17;11:485. doi: 10.3389/fphar.2020.00485 (PMC7181472; doi:10.3389/fphar.2020.00485)
Supplement: Supplementary file 2 [file DataSheet_2.docx]

Supplementary Material 2

# Supplementary Data

Kunxian Capsule is made up of 3.3g *Tripterygium Hypoglaucum Hutch* (Kun Ming Shan Hai Tang), 3.3g *Epimedium* (Yin Yang Huo), 1.6g *Cuscutae Semen* (Tu Si Zi) and 1.6g *Lycii Fructus* (Gou Qi Zi).

There is no data of *Tripterygium Hypoglaucum Hutch* in BATMAN-TCM, so we use PubChem_ID to retrieve predicted targets in BATMAN-TCM. The compounds of the herbs are summarized in table 1 to table 4.

Table 1. The Compounds of *Tripterygium Hypoglaucum Hutch* (Kun Ming Shan Hai Tang)

| Compounds name | PubChem_ID |
| --- | --- |
| triptriolide | 58636974 |
| tripterolide | 5281664 |
| triptolide | 107985 |
| triptophenolide | 173273 |
| neotriptophenolide | 133766 |
| triptobenzene K | 101936047 |
| triptonolide | 46176013 |
| triptonoterpene methyl ether | 15011611 |
| triptobenzene H | 53363843 |
| triptonoterpene | 15011611 |
| triptoditerpenic acid | 192372 |
| triptoditerpenic acid B | 192372 |
| triptobenzene A | 15762008 |
| triptobenzene D | 101712250 |
| hypodiolide A | 72369 |
| triptoquinone A | 132524 |
| triptoquinone B | 11724191 |
| triptoquinone H | 101062652 |
| wilforlide A | 158477 |
| triptotriterpenic acid A | 127707 |
| triptocallic acid D | 44575705 |
| triptocallic acid C | 101937091 |
| 3-epikatonic acid | 10434225 |
| mesembryanthemoidigenic acid | 21594136 |
| canophyllal | 12302400 |
| friedelin | 91472 |
| 29-hydroxyfriedelan-3-one | 133561848 |
| polpunonic acid | 129317347 |
| celastrol | 122724 |
| celastolide | 101936042 |
| triptohypol A | 101936043 |
| wilforic acid A | 101707492 |
| triptohypol B | 101936044 |
| triptohypol C | 10411574 |
| wilforol A | 10096097 |
| wilforol B | 10366522 |
| demethylzeylasteral | 10322911 |
| wilforic acid C | 101707494 |
| cangoronin | 101616678 |
| hypoglaside A | 147938 |
| triptohypol D | 101062653 |
| triptohypol E | 44559123 |
| triptohypol F | 91895434 |
| hypodiol | 101936045 |
| salaspermic acid | 44593364 |
| hypoglaunine F | 102066433 |
| hypoglaunine E | 71717032 |
| 2-*O*-deacetyleuonine | 122198189 |
| wilfortrine | 73321 |
| wilforgine | 91895385 |
| wilfordine | 442556 |
| tripfordine C | 101416498 |
| palmitic acid | 985 |
| fumaric acid | 444972 |
| stearic acid | 5281 |
| tricosanoic acid | 17085 |
| stigmast-4-en-3-one | 5484202 |
| daucosterol | 5742590 |
| β-sitosterol | 222284 |
| *L*-epicatechin | 12309509 |
| catechin | 9064 |
| regelidine | 13968328 |
| evonine | 477609 |
| euonymine | 477607 |
| hypoglaunine C | 44584754 |
| hypoglaunine B | 44583769 |
| forrestine | 477605 |
| neoeunoymine | 477608 |
| hyponine E | 44583772 |
| hyponine D | 44593669 |
| wilfornine | 188698 |
| wilformine | 44584752 |
| wilfordinine C | 101018699 |
| wilfordinine B | 101018698 |
| wilfordinine A | 101018697 |
| triptonine A | 5481550 |
| wilforine | 601100 |
| hypoglaunine C | 100953353 |
| peritassine A | 131676056 |
| triptonediol | no data in PubChem |
| 1β-benzoyl-8α-cinnamoyl-4α,5α-dihydroxydihydroagarofuran | no data in PubChem |
| triptobenzene J | no data in PubChem |
| triptobenzene L | no data in PubChem |
| quinone 21 | no data in PubChem |
| 3-acetoxy oleanolic acid | no data in PubChem |
| glut-5-en-3β,28-diol | no data in PubChem |
| 3-oxo-olean-Δ^9(11),12^-diene | no data in PubChem |
| 3-oxo-oleanoic acid | no data in PubChem |
| oleanoic acid 3-*O*-acetate | no data in PubChem |
| 2,3-dihydroxy-6-oxo-D:A-froedo-24 nor-1,3,5 (10),7-oleanatetraen-29-oic acid | no data in PubChem |
| populnilic acid | no data in PubChem |
| 3β-acetoxy-urs-12-ene-28-oic acid | no data in PubChem |
| 23-nor-oxopristimerol | no data in PubChem |
| 23-nor-6-oxo-demethyl pristimerol | no data in PubChem |
| 1α-acetoxy-6β,9β-dibenzoyloxy-4β-hydroxy-dihydroagarofuran | no data in PubChem |
| 3,4,5-trimethoxyphenyl-β-*D*-glucopyranoside | no data in PubChem |
| 3,4-dimethoxyphenyl-β-*D*-glucopyranoside | no data in PubChem |
| 3-methoxy-4-hydroxy-benzoic acid | no data in PubChem |
| 3,4-dihydroxy-benzoic acid | no data in PubChem |
| *p*-hydroxyl benzoic acid | no data in PubChem |
| procyanidin B-2 | no data in PubChem |
| procyanidin B-4 | no data in PubChem |
| procyanidin B-3 | no data in PubChem |
| ergosta-4,6,8 (14),22-tetraen-3-one | no data in PubChem |
| (2*R*,3*R*)-3,5,7,3′,5′-pentahydroxyflavan | no data in PubChem |
| 4′-*O*-(−) methylepigallocatechin | no data in PubChem |
| 3-pyridinecarboxylic acid | no data in PubChem |
| cangorinine E-I | no data in PubChem |
| hyponine C | no data in PubChem |
| hyponine B | no data in PubChem |
| hyponine A | no data in PubChem |
| hypoglaunine D | no data in PubChem |
| hyponine F | no data in PubChem |
| triptonine B | no data in PubChem |

Table 2. The Compounds of *Epimedium* (Yin Yang Huo)

| Compounds name | PubChem_ID |
| --- | --- |
| Magnoflorine | 73337 |
| Baohuoside I | 5488822 |
| Kaempferol | 5280863 |
| Epimedin A | 92043273 |
| Yinyanghuo B | 5315394 |
| Hentriacontane | 12410 |
| Ikarisoside B | 44258785 |
| Yinyanghuo A | 5315393 |
| Yinyanghuo D | 5315396 |
| Yinyanghuo E | 5315397 |
| Hyperin | 5281643 |
| Epimedokoreanoside I | 87759921 |
| Stearin | 11146 |
| Yinyanghuo C | 5315395 |
| Epimedin C | 5748394 |
| Icariin | 5318997 |
| Hyperoside | 5281643 |
| Wushanicariin | 3082728 |
| Epimedoside A | 44259072 |
| Ikarisoside F | 13964061 |
| Epimedin B | 5748393 |
| Magnograndiolide | 5319198 |
| Baohuoside Vi | 5488005 |
| Linoleyl Acetate | 21159087 |
| Epimedoside C | 44258783 |
| Epimedokoreanoside Ii | 44259071 |
| Wuweizi Alcohol B | 634470 |
| Kaempferitrin | 5486199 |
| Baohuoside Ii | 5481982 |
| Bilobanol | no data in PubChem |
| Icaride A2 | no data in PubChem |
| Diphylloside A | no data in PubChem |
| Yixinoside A | no data in PubChem |
| 20-Hexadecanoylingenol | no data in PubChem |
| Des-O-Methylicariin | no data in PubChem |
| Hentriacontanol-6 | no data in PubChem |
| Icariside A7 | no data in PubChem |

Table 3. The Compounds of *Cuscutae Semen* (Tu Si Zi)

| Compounds name | PubChem_ID |
| --- | --- |
| Campesterol | 173183 |
| Neosurugatoxin | 46173822 |
| Hyperoside | 5281643 |

Table 4. The Compounds of *Lycii Fructus* (Gou Qi Zi)

| Compounds name | PubChem_ID |
| --- | --- |
| Beta-Carotene | 5280489 |
| Tryptophan | 6305 |
| Cycloartanol | 12760132 |
| Lupeol | 259846 |
| Scopolin | 439514 |
| Atropine | 174174 |
| Vitamin B1 | 6042 |
| Cholesteryl Ferulate | 22375086 |
| Cyclofoetoside B | 441917 |
| Cinnamic Alcohol | 5315892 |
| Physcion-8-O-Beta-D-Gentiobioside | 442762 |
| Safranal | 61041 |
| Cholesterol | 5997 |
| Citrulline | 9750 |
| Campesterol | 173183 |
| Stigmasterol | 5280794 |
| Taurine | 1123 |
| Glycine | 750 |
| Scopoletin | 5280460 |
| Zeaxanthin | 5280899 |
| Trans-Cinnamic Acid | 444539 |
| Vitamin B2 | 493570 |
| Beta-Amyrin | 73145 |
| Vitamin B12 | 5311498 |
| Lantadene A | 6436598 |
| Ascorbic Acid | 54670067 |
| Vitamin B5 | 6613 |
| Tr-Saponin A | 56663921 |
| Cycloeucalenol | 101690 |
| Betaine | 247 |
| Hypaconitine | 441737 |
| Obtusifoliol | 65252 |
| Zederone | 101286196 |
| Delta-Carotene | 5281230 |
| Physalin A | 12314218 |
| Safrole | 5144 |
| Lupeol Acetate | 92157 |
| Gamma-Aminobutyric Acid | 119 |
| Nicotinic Acid | 938 |
| Gamma-Sitosterol | 457801 |
| Vitamin C | 54670067 |
| Citrostadienol | 9548595 |
| Hyoscyamine | 154417 |
| Cycloartanol Acetate | 537304 |
| Ipolamiide | 442425 |
| Glycitein | 5317750 |
| Physalien | 5281250 |
| 24-Methylenelophenol | 5283640 |
| Lanosterol | 246983 |
| Carotene | 6419725 |
| Riboflavine | 58683 |
| (24r)-4alpha-Methyl-24-Ethylcholesta-7,25-Dien-3beta-Yl Acetate | no data in PubChem |
| 24-Methylene Cycloartan-3beta,21-Diol | no data in PubChem |
| 31-Norcyclolaudenol | no data in PubChem |
| 24-Methylene Cycloartanol Ferulate | no data in PubChem |
| 12-O-Nicotinoylisolineolone | no data in PubChem |
| Cryptoxanthin Monoepoxide | no data in PubChem |
| 24-Methyl-31-Norlanost-9(11)-Enol | no data in PubChem |
